# Transstadial Transmission and Long-term Association of Crimean-Congo Hemorrhagic Fever Virus in Ticks Shapes Genome Plasticity

**DOI:** 10.1038/srep35819

**Published:** 2016-10-24

**Authors:** Han Xia, Andrew S. Beck, Aysen Gargili, Naomi Forrester, Alan D. T. Barrett, Dennis A. Bente

**Affiliations:** 1 Department of Microbiology and Immunology, University of Texas Medical Branch, Galveston, TX, USA; 2 Galveston National Laboratory, Galveston TX, USA; 3 Wuhan Institute of Virology, Chinese Academy of Sciences, Wuhan, China; 4 Department of Pathology, and Sealy Center for Vaccine Development, University of Texas Medical Branch, Galveston, TX, USA; 5 Marmara University, Istanbul, Turkey

## Abstract

The trade-off hypothesis, the current paradigm of arbovirus evolution, proposes that cycling between vertebrate and invertebrate hosts presents significant constraints on genetic change of arboviruses. Studying these constraints in mosquito-borne viruses has led to a new understanding of epizootics. The trade-off hypothesis is assumed to be applicable to tick-borne viruses too, although studies are lacking. Tick-borne Crimean-Congo hemorrhagic fever virus (CCHFV), a member of the family *Bunyaviridae*, is a major cause of severe human disease worldwide and shows an extraordinary amount of genetic diversity compared to other arboviruses, which has been linked to increased virulence and emergence in new environments. Using a transmission model for CCHFV, utilizing the main vector tick species and mice plus next generation sequencing, we detected a substantial number of consensus-level mutations in CCHFV recovered from ticks after only a single transstadial transmission, whereas none were detected in CCHFV obtained from the mammalian host. Furthermore, greater viral intra-host diversity was detected in the tick compared to the vertebrate host. Long-term association of CCHFV with its tick host for 1 year demonstrated mutations in the viral genome become fixed over time. These findings suggest that the trade-off hypothesis may not be accurate for all arboviruses.

Crimean-Congo hemorrhagic fever (CCHF) is an acute, often fatal viral hemorrhagic fever in humans but there are no licensed vaccines or antivirals available to prevent the disease. The causative agent, CCHF virus (CCHFV), is a member of the genus *Nairovirus*, family *Bunyaviridae*, and is an arthropod-borne virus (arbovirus) that has both tick and mammalian hosts^1^,^2^. The viral genome encompasses 3 segments, small (S), medium (M), and large (L). Each of the three genome segments contains a single open reading frame (ORF) flanked by noncoding regions, encoding the nucleocapsid (NP), the membrane glycoprotein precursor (GPC), and RNA-dependent RNA polymerase (RdRp), respectively. Five known proteins (Mucin-like domain, GP38, Gn, Nsm, and Gc) are generated from the glycoprotein precursor^3^. Strikingly, genomic sequencing has shown that CCHFV displays great genetic diversity among strains. Nevertheless, most of the sequences available come from cell culture passaged isolates. Previous studies have shown that certain areas of the CCHFV genome, such as the mucin-like domain or NSm, exhibit features of high genetic flexibility^4^,^5^.

CCHFV is maintained and transmitted in a silent vertical and horizontal transmission cycle involving the predominant tick vector *Hyalomma* spp. and a variety of small feral and domestic vertebrate species^6^. Epidemiological data indicate that CCHFV is only transiently associated with the vertebrate host and persists in ticks for the duration of their lifespan through transstadial transmission. CCHFV can also be transmitted vertically to the next generation^7^,^8^. As a result, ticks are thought to be not only the vector, but also an excellent long-term reservoir for the virus^7^,^9^,^10^. Furthermore, overwintering of infected tick life stages can further extend the association of the virus with the tick host, potentially for months or years, and this is hypothesized to play a critical role in the maintenance of epidemic foci. Nevertheless, no studies have been conducted to investigate what influence the transstadial transmission and long-term association has on viral evolution and its potential for transmission^7^,^10^,^11^. Since CCHFV is highly pathogenic, it must be studied under maximum biocontainment (biosafety level 4 [BSL4]) and most work performed on CCHFV involves investigation of genomic sequences isolated from infected humans and those available from ticks collected off animal hosts^5^,^12^. This has given us an understanding of the ecology and phylogenic divergence patterns of CCHFV^5^. However, these studies only present a snapshot of the virus evolution and there have been no studies on how the virus-host interaction in the different phases of the virus life cycle shapes viral evolution over time^13^,^14^.

RNA viruses have high mutation rates due to the lack of proof-reading by the RNA dependent RNA polymerase, which together with rapid replication kinetics can generate great genetic diversity compared to other viral pathogens^15^,^16^. Lower levels of genetic diversity are frequently observed for arthropod-borne RNA viruses compared to other RNA viruses, because the genomes of arthropod-borne RNA viruses need to maintain productive infection in both arthropod and vertebrate host cells. Maintenance of the viral lifecycle on two fronts is speculated to constrain the evolutionary processes acting on arbovirus genomes, and this has been termed the “trade-off” hypothesis^17^. The trade-off hypothesis proposes that cycling between different vertebrate and invertebrate hosts presents significant constraints on genetic change of arthropod-borne viruses, in part because of the dual effect of bottlenecks and selective pressure, and has become a central paradigm in understanding the forces that shape arbovirus evolution. To date, this hypothesis has been almost exclusively developed based on studies of mosquito-borne viruses, in particular the flaviviruses West Nile virus^18^ and St. Louis encephalitis virus^19^. However, the transmission cycle of tick-borne viruses differs significantly from mosquito-borne viruses in key features, such as transstadial and vertical transmission that play a chief role in virus perpetuation in nature.

Here, we investigate how the tick and the animal host shape the genome plasticity of CCHFV by studying the virus in a tick-mouse transmission cycle model. To study if there is genetic change during the infection of mice despite repeated passaging of the stock in vertebrate cells, mice were infected with CCHFV and sampled at the terminal phase of the disease. To study both the transmission from the mouse to the tick, as well as the transmission from one tick life stage to the next (transstadial transmission), *Hyalomma marginatum* ticks were fed on the mice that had been infected with CCHFV, and ticks were allowed to complete feeding and molt to the next life stage. Using next generation sequencing (NGS) as a tool to profile the CCHFV genome in both the tick and murine hosts, we detected a substantial number of nonsynonymous mutations in the tick host after only a single transstadial transmission that did not occur in CCHFV recovered from the mammalian host. Furthermore, greater viral intra-host diversity was detected in the tick compared to the vertebrate host, especially within the viral glycoprotein genes. We also investigated how the long-term association of the tick and the virus observed in nature shapes the CCHFV genome plasticity. To mimic the long-term association, some CCHFV-infected ticks were maintained for one year after molt before sampling. When we compared newly molted to one-year-old ticks, similar CCHFV titers were observed in both groups. Furthermore, we observed that the mutations in the CCHFV genome became fixed over time. These findings give new insight in the transmission of tick-borne viruses and suggest that our understanding of the trade-off hypothesis may not be accurate for all arthropod-borne viruses, at least as demonstrated by CCHFV.

## Results

### CCHFV is transmitted from the mammalian host to the tick and transstadially from the nymphal stage to the adult stage of the tick

**Figure 1:**
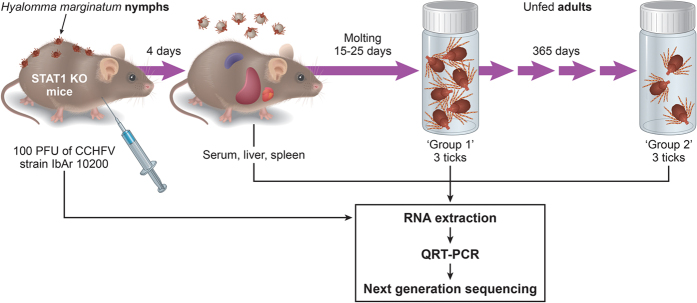
Experimental infection and sample collection. Naïve *Hyalomma marginatum* nymphs were allowed to feed on STAT1 KO mice for two days. Mice were infected with 100 PFU CCHFV IbAr 10200 after attachment. Nymphs were fully engorged by day 4 post challenge, when mice became moribund, and were allowed to molt to the adult stage. Liver, spleen, and serum were collected from mice. Three adult ticks (group 1) were used immediately after they completed molting (20 ± 5 days), and an additional three ticks were kept for 365 days after molt was completed (group 2). Illustration by James A. Perkins.

Immunocompetent mice don’t display clinical signs or viremia when challenged with CCHFV, therefore, STAT1 KO mice were infected with 100 PFU of CCHFV strain IbAr 10200, a model we previously described^20^, and showed clinical signs of infection beginning on day 3 and reached the predefined humane endpoint on day 4 post inoculation. As previously described^20^, high viral titers were detected in serum (10^9.63 to 10^10.13 genome equivalents (GEQ)/ml), liver (10^8.50 to 10^8.97 GEQ/μg RNA), and spleen (10^7.20 to 10^7.50 GEQ/μg) of mice (Fig. 1). All *Hyalomma marginatum* nymphs fed to completion on mice as shown by engorgement and ticks falling off the mouse host and molted to the adult life stage. CCHFV infection of nymphs and transstadial transmission was demonstrated in 10 out of 15 adult ticks (67%) feeding on CCHFV-infected mice by qRT-PCR detection of the viral genome. For our NGS analysis, 6 out 10 positive ticks were randomly selected: Group 1- three adult ticks were analyzed after they completed molting (20 ± 5 days); Group 2- three additional adult ticks were kept for 365 days after molt was completed. Titers ranged from 10^3.92 to 10^5.13 GEQ per tick in newly molted adult ticks (group 1) to 10^3.41 to 10^5.05 GEQ per tick in 1-year-old ticks (group 2). Titers in group 1 were not statistically different than in group 2 when analyzed by Student t-test (p = 0.8015) indicating that there is probably not a significant increase or decrease of virus replication over time.

### Full genome coverage of CCHFV is achieved by NGS

PCR amplicons spanning the full genome were generated for all tick samples and all murine tissues analyzed (excluding liver because two out of the seven amplicons in the L segment could not be generated for liver samples; therefore, liver samples were excluded from further studies). The deep sequencing analysis produced high quality paired-end reads for all samples. After removing the duplicated reads, each sample had considerable depth of coverage (Fig. S1); the average coverage per site for each sample was larger than 4,300-fold for the S segment, 6,300-fold for the M segment, and 5,800-fold for the L segment. The consensus sequences derived from the NGS data of the S and M segment of the CCHFV stock (strain IbAr 10200) used to infect the mice exactly matched the consensus Genbank reference sequences obtained by Sangar sequencing. However, eight nucleotide differences were observed in the L segment at positions 3,216 (G → A), 4,117 (C → A), 5,054 (C → A), 5,065 (U → A), 5,100 (C → A), 5,798 (C → U), 5,830 (A → U), and 5,875 (U → C) when compared to the reference sequence. Five of them were synonymous and three were nonsynonymous, which included sites in the RdRp at amino acid positions G1047E, P1660T, and T1675N. Thus, the consensus sequences obtained from the NGS results provided confidence in interpreting the diversity in the RNA population. Subsequent analyses were performed using the actual consensus sequence of the inoculum stock virus determined here compared to the sequences of viruses from mice and ticks.

### Comparison of CCHFV consensus sequences reveals mutations are found only in samples from tick vector but not in the mammalian host

The consensus sequences from the murine samples (serum, spleen), tick group 1 (newly molted ticks 1a, 1b, 1c), and tick group 2 (1 yr. old ticks; ticks 2a, 2b, 2c) were generated as described in the Material and Methods section, and the consensus sequences from each sample in mammalian host and tick vector were compared to the consensus sequence of the inoculum.

The consensus sequences of the murine serum and spleen samples exactly matched the inoculum virus sequence and all the consensus changes were found in the tick samples only. Fourteen consensus-level mutations, one in the S segment, four in the M segment, and nine in the L segment, were present in one or more tick samples and are summarized in Table 1. Almost all of the mutations occurred in nucleotide sites of the CCHFV genome where there is variability when consensus level sequences of isolates are compared to each other at that site (Table 1, right column). In newly molted ticks (group 1), all the ticks showed identical S segments to the consensus sequence. The consensus sequence of ticks 1b and 1c were identical and exhibited three nucleotide differences (position 1,142, 2,923, and 3,565) in the M segment, and six differences (position 1,495, 1,879, 8,896, 9,764, 11,374, and 11,761) in the L segment from that of the virus in mice; additionally, tick 1a had three additional nucleotide differences (position 1,142 in the M segment, and 2 differences (position 3,340 and 8,232) in the L segment) from the virus in mice. In the 1-year old ticks (group 2), the S segment of tick 2a was identical to the virus in mice, while a consensus difference from the mouse virus was observed for tick 2a at position 665 in the M segment, and four differences (position 1,495, 1,879, 8,896, and 9,764) in the L segment. For tick 2b, there was one difference (position 1,000) in the S segment, three differences (position 1,142, 2,923, and 3,565) in the M segment, and seven (position 1,495, 1,879, 5,305, 8,896, 9,764, 11,374, and 11,761) in the L segment. There were eight differences for tick 2c, one difference in the S segment, three differences (position 1,142, 2,923, and 3,565) in the M segment, and four differences (position 1,495, 1,879, 8,896, and 9,764) in L segment.

Notably, one noncoding mutation located in the M segment at nucleotide 1,142 (A → C) was shared in each individual tick in groups 1 and 2, except tick 2a; however, it was present in 45.97% of the reads in tick sample 2a and therefore close to consensus-level. This mutation was not present in the mouse samples. Moreover, four nucleotide mutations at positions 1,495 (C → U), 1,879 (A → G), 8,896 (G → A), and 9,764 (G → A, RdRp-V3230I) in the L segment were present in each sample in the 1-year-old ticks (group 2), but were not found in the newly molted ticks (group 1). However, a high number of single nucleotide variants (see below) were observed in the same sites in group 1 ticks.

Four of the fourteen mutations were nonsynonymous, resulting in amino acid substitutions at sites 2,923 (NSm-K944T) and 3,565 (Gc-G1158E) in the M segment, and sites 8,232 (RdRp-S2719F) and 9,764 (RdRp-V3230I) in the L segment.

### Distribution of single nucleotide variants across the genome differs in mammalian host vs the tick vector

Single nucleotide variants (SNVs) in viral RNAs extracted from the mammalian host and tick vector were analyzed using VarScan and Geneious software.

**Figure 2:**
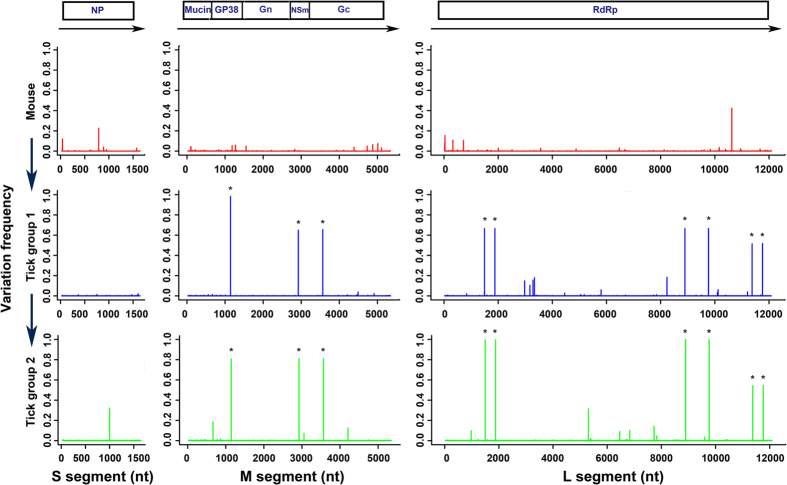
Overview of the variation frequency at each position of CCHFV genome in different host groups compared to the viral stock. A peak stands for frequency (the mean value within group) of variants at each site (red, mouse group, n = 2; blue, tick group 1, n = 3; green, tick group 2, n = 3). The bar with asterisk indicates the consensus level mutation. The boxes at the top of the figure represent open reading frames (ORFs) corresponding to the NP, Mucin, GP38, Gn, Nsm, Gc, and RdRp proteins. Coding directions are indicated by the black arrows.

**Figure 3:**
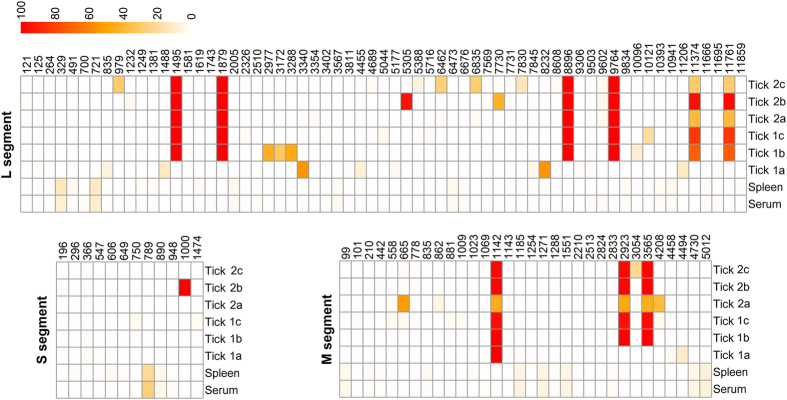
Heat map showing the distribution of the SNVs (frequencies >1%) for all samples from mammalian and tick hosts. The colored bar indicates the percentage of the variation (red equals 100%, and the white equals 0). Newly molted ticks are designated 1a, 1b, and 1c, while 1 yr. old ticks are labeled 2a, 2b, and 2c.

A total of 107 SNVs were identified at a frequency of greater than 1.00 percent of the population from one or more samples from the mammalian and tick host. Comparison of the mean variation frequency per site (>1%) of each sample with the other samples within the same group showed there was no statistically significant difference. Thus, the samples were combined and analyzed as a group. When the mean variation frequency per site (>1%) for each group (murine samples vs tick group 1 vs tick group 2) was compared, more variant sites were detected in the S and M segments in each of the mouse samples compared to the tick samples. However, more variant sites in L segments were detected in the tick samples of group 1 and 2 compared to mouse samples (Fig. 2). Variant sites (M1142, M2923, M3565, L1495, L1879, L8896, L9764, L11374, and L11761) were found in at least 5 out of 6 ticks, indicating that this occurrence is not a random process (Fig. 3). When analyzed by Fisher exact test, there was a statistical significant difference in the SNV numbers in the M segment (mouse vs tick group 1, p = 8.243e-06; mouse vs tick group 2, p = 8.243e-06), however, the difference in SNV numbers were not statistically significant for the S segment (mouse vs tick group 1, p = 0.274; mouse vs tick group 2, p = 0.018) or the L segment (mouse vs tick group 1, p = 0.116; mouse vs tick group 2, p = 0.149). A similar trend was observed when the nonsynonymous mutations were examined by Fisher exact test: there was a statistical significant difference in the M segment between the mammalian and tick groups (mouse vs tick group 1, p = 0.0029; mouse vs tick group 2, p = 0.0029), however, there was no difference in the S segment (mouse vs tick group 1, p = 1; mouse vs tick group 2, p = 0.067) and L segment (mouse vs tick group 1, p = 0.1528; mouse vs tick group 2, p = 0.1035). These findings emphasize that there might be key differences in the replication of the M segment in the two hosts.

Interestingly, when SNVs were analyzed at a higher-frequency polymorphisms cut-off (>5%) greater SNV numbers in L segment were seen in the tick versus mammalian sample groups (Fig. 3).

### Whole CCHFV genome nucleotide diversity is greater in the tick than in the mammalian host

**Figure 4:**
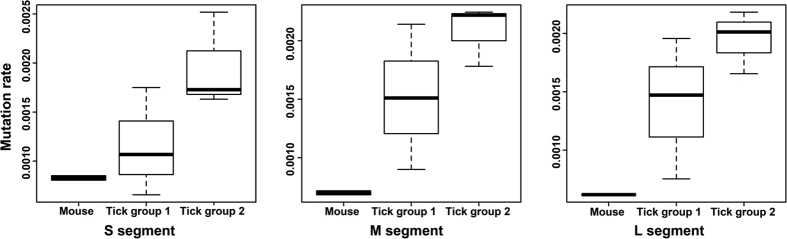
Boxplot for the mutation rate of each segment for CCHFV in mammalian and tick hosts. The Kruskal-Wallis test was used to test for significant difference in the mutation rate of the tick and mouse groups in all 3 segments (S segment, p = 0.2082; M segment, p = 0.0685; L segment, p = 0.0685).

To investigate nucleotide diversity, the mutation frequency of each CCHFV segment were determined for each sample as described in the Methods and mean values for each group were calculated (Fig. 4). A Kruskal-Wallis test was used to compare the difference of mutation frequency amongst groups since the data in tick group 2 was not normally distributed. Although a clear trend is apparent between the mouse and the tick samples (Fig. 4), there was no statistical significant difference between groups in all three segments. However, the p-value for the M and L segment were near to the predefined value of 0.05 (S segment, p = 0.2082; M segment, p = 0.0685; L segment, p = 0.0685).

### Purifying selection is greater in the mammalian host (dN/dS ratio)

**Figure 5:**
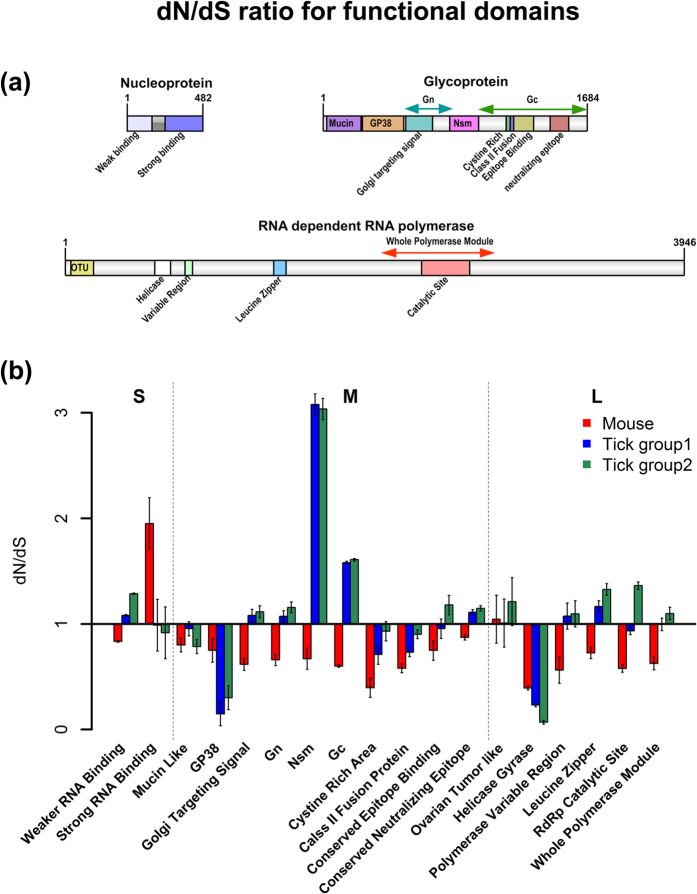
dN/dS ratio for each functional domain of CCHFV in mammalian and tick hosts. (**a**) the genomic structure of verified and proposed functional domain. (**b**) dN/dS ratio for each domain, dN/dS > 1: positive selection, dN/dS < 1: purifying selection, and dN/dS = 1: neutral selection. The error bar stands for s.d. value (n = 2 for mouse; n = 3 for tick group 1 and 2).

The ratio of nonsynonymous to synonymous differences (dN/dS) is widely used to estimate the role of selection pressure in the evolution of a protein coding gene in a population. The three RNA segments of CCHFV encode different viral proteins, and a total of 18 verified and putative functional domains were analyzed (Table S2). The dN/dS ratios were calculated for each domain (Fig. 5). In both mouse samples, purifying selection (dN/dS < 1) was observed in all of the functional domains, except the RNA binding domain located in the S segment and the ovarian tumor-like domain in the L segment (Fig. 5). In comparison, positive selection (dN/dS > 1) was most common throughout the functional domains in tick group 1 and group 2 samples with the exception was the GP38 domain in the M segment and the helicase/gyrase domain in the L segment, where negative selection was observed. Furthermore, the strongest positive selection was observed in the NSm domain in both of the tick groups.

## Discussion

Arboviruses are transmitted among vertebrate hosts by hematophagous arthropod vectors, such as mosquitoes, other biting flies, and ticks, and are the cause of disabling febrile syndromes in millions of people worldwide. Studies focusing on mosquito-borne viruses, in particular alpha- and flaviviruses^17^, have greatly improved our understanding of epizootics and emergence of vector-adaptive mutants. These studies have also lead to the development of the central paradigm of arbovirus evolution, the “trade-off” hypothesis, which proposes that cycling between highly divergent vertebrate and invertebrate hosts constrains genetic change of arboviruses. In comparison, there is a striking lack of analogous data available for tick-borne viruses and existing data have focused on flaviviruses only^19^,^21^. Nevertheless, transmission cycles of tick-borne viruses differ in key features from mosquito-borne viruses that play a major role in virus perpetuation, such as vertical and transstadial transmission. As a result, tick-borne viruses are significantly longer associated with their vector than mosquito-borne viruses. Tick-borne CCHFV displays a great degree of nucleotide sequence diversity with divergence of up to 20%, 31%, and 22% among the S, M, and L segments, respectively, of virus isolates when the consensus sequences of the entire segments are compared to each other^2^. However, only a very small percentage of CCHFV genome sequences in Genbank come directly from ticks but rather from isolates passaged in mammalian cell culture or suckling mouse brains. CCHFV’s broad genetic diversity contrasts with the much closer nucleotide sequence homology of other bunyaviruses, such as mosquito-borne Rift Valley fever virus (4% S segment; 5% M segment; 4% L segment), whose evolution appears to have been more constrained by the requirement to replicate in a few vertebrate and mosquito species^22^,^23^. Another major difference between tick and mosquito species is our understanding of the bottlenecks associated with viral infection^24^,^25^,^26^,^27^,^28^,^29^,^30^,^31^. It is well known that the mosquito host creates several bottlenecks for the virus as it replicates within the vector that significantly impact the diversity of the virus generated during infection. It is possible that like mosquitoes^32^,^33^,^34^ the initial infection and dissemination of virus in the ticks imposes bottlenecks. However, as we did not sample the ticks prior to molting due to the necessity to sacrifice the ticks for sampling, it is difficult to determine if bottlenecks play a critical role. Our results show that when following tick infection over a period of one year, there is directed selection for certain mutations that must be advantageous for CCHFV in the tick. The presence of these mutations does not necessarily preclude the presence of bottlenecks, but in our opinion bottlenecks are less likely than the directed selection observed in the ticks.

Our goal was to investigate how the transstadial transmission of CCHFV and the long-term association with the vector shapes the genomic plasticity of CCHFV. Utilizing a recently established experimental transmission model for CCHFV in the BSL4, as well as employing *Hyalomma marginatum* ticks, mice, and NGS to profile the CCHFV genome populations in both the tick and murine hosts, we studied the genomic plasticity of CCHFV, both at a consensus and subpopulation level.

In our model, we demonstrated that 67% of engorged nymphs that had fed on CCHFV-infected mice were infected as adults. Significantly, we detected CCHFV to similar titers in newly molted and 1-year-old ticks; therefore, confirming that CCHFV can indeed be associated with the tick host for a long period of time most likely by low-level, continuous replication. Interestingly, we were not able to amplify the L segment in the liver samples from the mice. This could be suggestive of defective interfering particle production in these tissues leading to packaging of an incomplete set of genome segments in the virions^20^,^35^,^36^. To get full coverage and high sequencing depth of viral sequences, the overlapping fragments were generated by PCR for NGS to obtain entire genomic sequences from samples^37^,^38^,^39^. In the PCR amplification step, lower cycles (≤20 cycles) were used to reduce amplification bias. Furthermore, a high fidelity proofreading polymerase was used to amplify the fragments. In the downstream analysis, the PCR duplicates were removed to increase the accuracy of alignment. The consensus sequence for the genome of the CCHFV stock used in this study determined here by NGS was identical to that in Genbank for the S and M segments, and had only eight nucleotide changes in the L segment. In addition to the CCHFV IbAr 10200 reference sequence used here, other laboratories have sequenced CCHFV IbAr 10200 and published in GenBank with very few nucleotide differences to the sequence used here. This strong identification gave us confidence that the sequence data obtained in this study were representative of the dominant CCHFV quasispecies population. While three samples per group (tick 1, tick 2, and mouse) are low, in part imposed by the constraints of maximum biocontainment, the high degree of sample homogeneity within the groups suggests that the data are reproducible and representative for each experimental condition. Furthermore, the sample size was sufficient to yield statistically significant results in multiple tests.

SNV analysis is a valuable tool to track the changes that viral genomes undergo during host transmission. Strikingly, there were 14 consensus-level mutations in the tick virus that occurred within a single transstadial transmission only but none were found in CCHFV recovered from the mammalian host. Almost all of these mutations occurred in nucleotide sites with high variability (see Table 1, right column), and furthermore, many of these changes were in viral genes that show high variability in their entirety such as the mucin-like domain or NSm. Furthermore, the fact that these mutations occurred in the ticks but not in the mammalian host indicates that the mutations arose during replication in the tick. During molting, many of the tick tissues, especially salivary glands and midgut, undergo extensive remodeling through absorption and rebuilding. Although information is lacking for CCHFV, studies with other tick-borne viruses have shown that viral titers typically peak during molting when tissues in the tick are absorbed and reconstituted^9^,^11^,^40^,^41^. Therefore, we hypothesize that transstadial transmission by the tick vector is a major determinant of the genetic diversity of CCHFV, and the molting phase is a key process in expanding CCHFV intra-host diversity. In nature, *H. marginatum* ticks are two-host ticks with larvae and nymphs feeding in succession on the same host, including molting on the host. This study looked at one transstadial transmission only (from the nymphal to the adult life stage). Thus, we do not know if mutations continue to accumulate with each transmission, or if they halt due to adaptation of the virus to the tick. Future studies in our lab will address this question. A non-synonymous change within Gc (nucleotide 3,565) was observed in 4 out of the 6 ticks. This is significant since there is no sequence variability at this site within the genomes of other CCHFV isolates (Table 1, right column). Additionally, a noncoding mutation located in the M segment at nucleotide 1,142 (A → C) not present in the mouse samples was found in all ticks, except tick 2a; however, this SNV represented 46% of the population and close to consensus-level. This in addition to the 4 SNVs that became fixed over time (see below) may be evidence for a tick vector adaptive mutation. These findings will be further investigated using the CCHFV reverse genetics system^42^.

There were four consensus level nucleotide mutation sites in all ticks from one-year-old ticks (group 2) that were not present in the newly molted ticks (group 1). Interestingly, there was a pattern of sub-consensus changes in these sites in the newly molted ticks identified by NGS (Fig. 3). This suggests that the mutations at these sites continue to be fixed over time even if, as suspected, very little viral replication occurs during molting. This may be evidence for a tick vector adaptive process and will be investigated further with a reverse genetics system.

To date, studies comparing genetic divergence of CCHFV in ticks and mammals are currently lacking^43^ and only two studies, looking at flaviviruses from the tick-borne encephalitis complex, have examined the inter-host variation of a naturally occurring tick-borne virus population^21^,^44^. Both studies investigated naturally infected field collected ticks. Brackney *et al.* did not find significant intra-host genetic diversity whereas Casati *et al.*, although limited since only a small region of the viral genome was sequenced, did find significant intra-host diversity. These publications together with our findings may indicate that intra-host genetic diversity might be virus-specific. Nevertheless, neither studies look at the viral population in the mammalian and tick host longitudinally nor used NGS to achieve high sequencing coverage. Our studies are the first to look at the intra-host variability of a tick-borne virus over time and in a controlled laboratory setting. Although just above being statistically significant when analyzed by Kruskal-Wallis test, there is a clear trend showing that the CCHFV genome nucleotide diversity, particularly the M segment, is greater in the tick than in the mammalian host after one transstadial transmission and increases over time in the tick. A greater sample size might have given a statically significant result and will be addressed in future studies. Our findings are consistent with the epidemiological data based on consensus sequencing showing greater genetic diversity of the M segment compared to the S and L segments^5^,^45^. The greater diversity of the M segment probably reflects the critical role of the glycoproteins Gn and Gc in the viral life cycle, including binding to cell-surface mammalian and tick receptors, tissue tropism, and vector competence^2^. We propose that the tick, and not the vertebrate host, is driving the genetic diversity in the M segment. This hypothesis is supported by the fact that CCHFV is only temporarily associated with the vertebrate host^2^. In addition, a study has demonstrated striking similarities between the tick vector phylogenies and nairovirus phylogenies supporting the concept virus–tick coevolution^46^. A similar pattern has also been observed for tick-borne flaviviruses^47^. Nevertheless, our study is limited by three factors: a) we only studied infection of ticks from a mammalian host, and not the diversity of CCHFV strains transmitted from ticks to the mammalian host; b) reference strain IbAr 10200 has been passaged multiple times in suckling mouse brains. The repeated passage in the mammalian host might have a confounding effect on the intra-host diversity seen in the mouse samples in this study. Future studies will address this by using low mammalian cell culture passage or tick cell culture passaged CCHFV strains. Unfortunately, the availability of those strains is very limited; c) a mouse host with a functional innate immune response might have imposed either a more selective or diversifying pressure on CCHFV. Unfortunately, CCHFV infection in immunocompetent mice is refractory and in our laboratory leads to greatly reduced transmission rates.

It will be vital to study the driving force behind the diversity observed here. Work with mosquito-borne viruses has shown RNA interference (RNAi) is the major innate immune pathway controlling mutational diversity of mosquito-borne viruses. Work with West Nile virus, for instance, has shown that regions of the WNV genome that are more intensely targeted by RNAi are more likely to contain point mutations compared to weakly targeted regions^48^. Very little is known about RNAi in ticks^49^. Future studies ought to address how far the RNAi response shapes the diversity of CCHFV in ticks.

Examination of the dN/dS ratio for each proposed functional domain of CCHFV showed that, as expected, the virus undergoes mostly negative selection in the mammalian host; however, significantly, mostly positive selection was observed in tick host, especially in tick group 2 at one-year post infection and the viral glycoprotein genes, in particular. This scenario is not consistent with the “trade-off” hypothesis, as greater viral intra-host diversity was detected in the tick after molt compared to the vertebrate host. While positive selection was observed in samples collected from ticks, we cannot exclude that this observation was the result of this extensively mammalian-adapted virus reverting to its ancestral (i.e. wild-type) genotype when introduced into ticks. Thus, if our observations are a result of ancestral reversion then there is a possibility that the trade-off hypothesis may still be applicable to tick-borne viruses. Genetic plasticity could be beneficial in the presence of changing environments of the tick and an important determinant in viral evasion of the host and vector immune response. Interestingly, by far the strongest positive selection was observed in the NSm domain in both of the tick groups. The function of CCHFV’s NSm remains unclear. In RVFV, NSm is recognized to function as virulence factors^50^; however, NSm is not required in mammalian cell culture for efficient virus replication, assembly, or maturation. Moreover, studies have demonstrated that the deletion of NSm greatly reduced the infection, dissemination, and transmission rates of the virus in *Aedes aegypti* mosquitoes and infection rates in *Culex quinquefasciatus* mosquitoes^51^. The strong positive selection in the tick host seen in this study might suggest that NSm plays a critical role in the infection and dissemination of CCHFV in the tick host.

Our findings suggest a unique selection pressure for CCHFV in the tick host and warrant a detailed evaluation of the trade-off hypothesis for tick-borne viruses. Such studies will improve fundamental understanding of the tick-virus-host interaction that shapes the genomic diversity, and ultimately the virulence and transmissibility of CCHFV.

Finally, there are a number of possibilities to explain the differences between this study and previous studies with regard to the trade-off hypothesis. The first is that we focused our studies on a tick-borne virus rather than a mosquito-borne virus. Second, we are investigating a natural virus isolate, while most of the published mosquito-borne virus studies utilized infectious-clone derived viruses. Third, we have studied a bunyavirus that has a tripartite genome, whereas the other studies have examined alphaviruses and flaviviruses with monopartite genomes. While there are a number of hypotheses to explain the different results, there is little doubt that more work is needed in this area to better understand the factors driving the evolution of arthropod-borne viruses.

## Material and Methods

### Ethics Statement

All animal studies were performed in strict accordance with the recommendations in the Guide for the Care and Use of Laboratory Animals of the National Institutes of Health. All mouse studies were performed at the University of Texas Medical Branch using protocols reviewed and approved by the University of Texas Medical Branch Institutional Animal Care and Use Committee. All studies were performed in a manner designed to minimize pain and suffering in infected animals.

### Ticks and Animals

*Hyalomma marginatum (H. marginatum*) ticks used in this study were obtained from the Insectary Services Division of the Galveston National Laboratory (UTMB, Galveston, TX) where a colony is maintained. Unfed nymphs used in the *in vivo* transmission model were generated by first letting larvae feed to completion on rabbits, then pulling off the engorged larvae from the rabbit host and letting them molt to the nymphal stage. Four to eight-week-old female STAT-1 knockout mice (129S6/SvEv-*Stat1*^*tm1Rds*^; Taconic, Germantown, NY) and male New Zealand white rabbits, >1.5 kg (Charles River, Wilmington, MA) were used for all experimentation. Rabbits were used to feed the larvae of *H. marginatum* by ear bag infestation and were housed in Allentown isolator cages with commercial diet and water provided *ad libitum*. Mice were housed in sterile isolator cages (Tecniplast, Buguggiate, Italy) with sterile diet and water *ad libitum*, and exposed to ticks using feeding capsules (see below).

### CCHFV Infection

Five unfed nymphs were placed into one feeding capsule that had been applied to STAT-1 KO mice housed in the ABSL2 using techniques already reported^47^. On third day after ticks were placed into feeding capsule, tick attachment was confirmed and animals were moved from Animal Biosafety Level (ABSL) 2 to the ABSL4. Due to biocontainment requirements tick attachment needs to be ensured before animals can be moved into ABSL4. At this point in time, ticks were anchored in the skin but have not started engorging. On the same day, animals were challenged intraperitoneally with 100 plaque forming units (PFU) of CCHFV strain IbAr 10200 as previously described^20^. CCHFV strain IbAr 10200 was obtained from the World Reference Collection of Emerging Viruses and Arboviruses at UTMB (WRCEVA, passaged 13 times in suckling mice and one time in Vero E6; Genbank sequences: NC005302, NC005300, and NC005301) and was passaged twice in SW-13 cells (ATCC, CCL-105) before use. On day 4 post challenge, mice reached predefined humane endpoint, were euthanized, and liver, spleen, and serum were collected (Fig. 1). Simultaneously, nymphs completed feeding and were collected from feeding capsule. Engorged nymphs were placed in sterile, clear plastic sample vials with a sterile paper strip, and mesh tops, and allowed to molt to the adult stage within plastic desiccators in climate-controlled environmental growth chambers (27 °C ± 1 °C day, 21 °C ± 1 °C, night; 80% ± 5% relative humidity, and 12 h light/dark cycle) (Fig. 1). Three adult ticks (Group 1 = Tick 1a, Tick 1b, Tick 1c) were used immediately after they completed molting (20 ± 5 days). Three additional adult ticks were kept for 365 days after completing molt (Group 2 = Tick 2a, Tick 2b, Tick 2c). All ticks were euthanized by freezing at −80 °C for 60 minutes.

### RNA extraction

Total RNA was extracted from virus inoculum, mouse serum, liver, and spleen, as well as from whole ticks. For cell supernatant and serum samples, 1 ml Trizol LS (Invitrogen, Carlsbad, CA) was added to the tube containing 200 ul of the sample and incubated for 10 min. For tick samples, individual ticks were weighed and then homogenized in 1 ml Trizol using yttria-stabilized zirconia beads (20 mm) in a Tissuelyser II system (Qiagen) for 4 min. Subsequently, cell debris was removed by centrifugation. For mouse tissues, 1 ml Trizol was added to a tube containing ≤50 mg of tissue, homogenized for 4 min using a single 50 mm metal bead (Tissuelyser II, Qiagen), and incubated for 10 min; cell debris was removed by centrifugation. RNA was isolated from homogenates by phenol/chloroform extraction with for qRT-PCR testing of CCHFV. All samples were tested for CCHFV and amounts calculated as genome equivalence by qRT-PCR with a recombinant RNA standard as previously described^52^, using Qiagen’s Quantitect Fast (Qiagen) probe kit and were run on a Roche Lightcycler real-time detection system (Roche).

### Next Generation Sequencing and Data Analysis

A total of nine samples (virus inoculum, mouse serum and spleen, three newly molted adult ticks, and three 1 yr. old adult ticks) were analyzed by deep sequencing. The detailed information for each sample is listed in Table S3.

First-strand cDNA was synthesized from the extracted RNA using random hexamers, following the instruction of Superscript III First-strand Synthesis kit (Invitrogen). The target cDNA was then amplified directly via PCR using Phusion High-Fidelity PCR kit (NEB) following the manufacturer’s protocols. The following PCR conditions were used: 98 °C for 1 min, followed by 20 cycles of 98 °C for 10 s, 55 °C for 30 s and 72 °C for 1 min, with a 10 min final extension at 72 °C. Eleven primer pairs were designed to produce overlapping (the overlap ranging from 150–400 bp) amplicons spanning the entire genome of CCHFV (Table S4), one of them targeted S segment, three to M segment, and seven to L segment. The 11 amplicons per viral sample were purified through the QIAquick PCR Purification Kit (Qiagen). The purified products were quantified using a BioAnalyzer 2100 platform (Agilent Technologies, Inc.) and then mixed and adjusted to equimolar ratios for each sample. Libraries were prepared using the Nextera®DNA kit (Illumina, San Diego, CA). Sequencing was performed on an Illumina HiSeq 1500 instrument, generating 50b paired-end, multiplexed reads.

### Quality trimming and alignments of Illumina data

Trimmed reads were filtered for quality scores (Q > 35). Three bases were trimmed from the 5′ ends of all reads. Alignments were performed with paired-end mode with the –S option to obtain the output in SAM format through Bowtie2 v2.2.5 (http://bowtie-bio.sourceforge.net/bowtie2/index.shtml)^53^. Nucleotide sequences of three viral segments of CCHFV IbAr10200 were retrieved from Genbank: NC005302 (nucleoprotein), NC005300 (glycoprotein precursor), and NC005301 (putative polyprotein). The IbAr 10200 reads were aligned to the Genbank reference sequences to generate the consensus sequences. All of the reads from the mouse and tick samples were aligned to the new consensus. Sam tools v1.2 (http://www.htslib.org/) was used to compress the sequence alignment files from SAM format to BAM format. Picard-tools v1.130 (http://broadinstitute.github.io/picard/) was used to remove PCR duplicate reads from read alignments. The original NGS data was deposited in the Sequence Read Archive (SRA) database, the SRA study accession number is SRP064987.

### Single Nucleotide Variants (SNV) and amino acid site changes detection

Consensus-level mutations were defined as polymorphisms that appeared in more than 50% of all the reads at a particular position; the consensus nucleotide was used as a reference for SNV calling. SNVs were called using VarScan2 v2.3.7 (http://varscan.sourceforge.net/), using a minimum base quality of 30, minimum coverage of 300, minimum variant frequency of 0.01, considering only those allele variants with more than eight supporting reads in both plus and minus strands were considered. Geneious v8.1 (Auckland, NZ) was used as an independent method of calling SNVs (same constraints) to ensure call reproducibility. Geneious variant calling occurred only at sites inside the coding regions with reads coverage >300, and variation frequency of 0.01. SNV annotation and counts of non-synonymous/synonymous changes at the amino acid site were performed by DiversiTools (http://josephhughes.github.io/btctools/). The consensus sequences for the reference strain IbAr 10200 were used as the search database to perform the SNV classification. The numbers of SNV (variation frequency >1%) and nonsynonymous mutation in 3 segments were counted from each group (mouse, tick group 1, tick group 2). Subsequently, a Fisher’s exact test was performed to analyze the relationship between (1) all SNVs with host, and (2) coding SNVs with host.

### Calculation of nucleotide diversity estimates and dN/dS ratio

The percentage of nucleotide mutations (total number of variation divided by the total number of sequenced nucleotides) was used as an estimate of genetic diversity^21^. The Kruskal-Wallis test was used to analyze the differences between group means of nucleotide diversity.

The dN/dS is the ration of the number of nonsynonymous substitutions per nonsynonymous site (p_N_) to the number of synonymous substitutions per synonymous site (p_S_), which can be used for investigating the role of selection in the evolution of a protein coding gene in a population. The ratio greater than one implies positive or Darwinian selection, less than one implies purifying selection, and a ratio of one indicates neutral selection.

The following formula takes into account the read coverage at each codon and was used to calculate p_N_ as well as p_S_ from the NGS data^54^,^55^,^56^. For each read (c_i_) covering a particular codon (r_i_), the observed number of nonsynonymous mutations in the read compared to the reference was calculated (n_dij_) and divided by the expected number (n_i_). The value for all reads at the codon is summed and averaged by coverage (c_i_). Then, the value for all codons is summed and divided by codons number (r) to give a single value for the whole ORF. Then the pN (likewise pS) was used to calculate the dN(dS) and the dN/dS ratio (equation (1)) (http://bioinformatics.cvr.ac.uk/blog/calculating-dnds-for-ngs-datasets/).


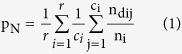


The DiversiTools (http://josephhughes.github.io/btctools/) was used to calculate the value for c_i_, n_i and_ n_dij._ Then the pN (likewise pS) was used to calculate the dN(dS) and the dN/ds ratio.

### Statistical analysis

A t-test was used to compare the mean GEQ between adult ticks (group 1) and 1-year-old ticks (group 2). The Fisher exact test was used to compare the difference in the numbers of SNVs and nonsynonymous mutations for each segment amongst mouse and tick groups. The Kruskal-Wallis test was used to compare the difference of mutation rate amongst mouse and tick groups. The t-test, Fisher, and Kruskal-Wallis test were conducted by the ‘stats’ package in the R v3.2.0 (http://www.r-project.org/).

## Additional Information

**How to cite this article**: Xia, H. *et al.* Transstadial Transmission and Long-term Association of Crimean-Congo Hemorrhagic Fever Virus in Ticks Shapes Genome Plasticity. *Sci. Rep.* **6**, 35819; doi: 10.1038/srep35819 (2016).

## Supplementary Material

Supplementary Information

## Figures and Tables

**Table 1:** Consensus-level mutations and their characterization.

Segment	Position	Mutation	Gene	Verified Domain	Syn/Nonsyn	Frequency in Sample (%)								Nucleotide similarity per site among all CCHFV isolates^b^
						Mouse		Tick group 1			Tick group 2			
						Serum	Spleen	1a	1b	1c	2a	2b	2c	
S	1,000	C->A	Nucleoprotein		Syn	0.02	0.02	0.02	0.08	0.01	0.46	96.2^a^	0.03	>80%
M	665	G->A	Glycoprotein	Mucin	Syn	0.04	0.04	0.07	0.06	3.55	52.8^a^	0.89	1.97	60–80%
	1,142	A->C	Glycoprotein	GP38	Syn	0.02	0	99.87^a^	99.90^a^	95.43^a^	45.79	98.65^a^	97.99^a^	<60%
	2,923	A->C	Glycoprotein	Nsm	Lys->Thr	0.03	1.09	0.22	99.78^a^	95.08^a^	47.46	98.60^a^	97.54^a^	<60%
	3,565	G->A	Glycoprotein	Gc	Gly->Glu	0.02	0	0.03	99.94^a^	96.94^a^	44.97^a^	99.10^a^	98.84^a^	100%
L	1,495	C->T	Polymerase		Syn	0	0.02	0	100^a^	99.94^a^	99.99^a^	99.97^a^	99.85^a^	<60%
	1,879	A->G	Polymerase		Syn	0.01	0	0.03	100^a^	100^a^	99.93^a^	100^a^	99.99^a^	<60%
	3,340	T->C	Polymerase		Syn	0.04	0	54.82^a^	0.03	0.01	0.01	0	0	<60–80%
	5,305	G->A	Polymerase		Syn	0.02	0.02	0.02	0.04	0.11	0.16	94.82^a^	0.03	>80%
	8,232	C->T	Polymerase	RdRp core	Ser->Phe	0.01	0.03	55.29^a^	0.01	0.04	0	0.03	0.03	100%
	8,896	G->A	Polymerase		Syn	0.03	0.02	0.06	99.94^a^	99.93^a^	99.97^a^	99.98^a^	99.96^a^	<60%
	9,764	G->A	Polymerase		Val->Ile	0.01	0.21	0.01	99.98^a^	99.97^a^	99.98^a^	99.98^a^	99.96^a^	<60%
	11,374	G->A	Polymerase		Syn	0.01	0	0.03	71.01^a^	83.61^a^	38.30^a^	96.62^a^	29.05	>80%
	11,761	C->T	Polymerase		Syn	0.04	0.20	0.01	72.17^a^	83.15^a^	38.59	97.31^a^	28.39	<60%

^a^Consensus-level mutations.

^b^GenBank published sequences for S- (111 sequences), M- (82 sequences), and L- (71 sequences) genomic segments were aligned using Geneious Software 9.1.5 (Biomatters, Ltd., Auckland, New Zealand) using global alignment criteria with free end gaps and a cost matrix of 65% similarity. Similarity per nucleotide site were analyzed with respect to the consensus sequence of CCHFV strain: IbAr10200. Similarities of nucleotides to IbAr10200 were notated as being <60%, 60–80%, >80%, or 100% similar in the alignment.

## References

[b1] Whitehouse C. A. Crimean-Congo hemorrhagic fever. Antiviral Res. 64, 145–160 (2004). 15550268 10.1016/j.antiviral.2004.08.001

[b2] Bente D. a. *et al.* Crimean-Congo hemorrhagic fever: History, epidemiology, pathogenesis, clinical syndrome and genetic diversity. Antiviral Res. 100, 159–189 (2013). 23906741 10.1016/j.antiviral.2013.07.006

[b3] Bergeron E., Vincent M. J. & Nichol S. T. Crimean-Congo hemorrhagic fever virus glycoprotein processing by the endoprotease SKI-1/S1P is critical for virus infectivity. J. Virol. 81, 13271–13276 (2007). 17898072 10.1128/JVI.01647-07 PMC2169102

[b4] Hewson R. *et al.* Crimean-Congo haemorrhagic fever virus: Sequence analysis of the small RNA segments from a collection of viruses world wide. In Virus Research 102, 185–189 (2004). 15084400 10.1016/j.virusres.2003.12.035

[b5] Deyde V. M., Khristova M. L., Rollin P. E., Ksiazek T. G. & Nichol S. T. Crimean-Congo hemorrhagic fever virus genomics and global diversity. J. Virol. 80, 8834–8842 (2006). 16912331 10.1128/JVI.00752-06 PMC1563879

[b6] Ergonul O. Crimean-Congo haemorrhagic fever. Lancet Infectious Diseases 6, 203–214 (2006). 16554245 10.1016/S1473-3099(06)70435-2 PMC7185836

[b7] Hoogstraal H. The epidemiology of tick-borne Crimean-Congo hemorrhagic fever in Asia, Europe, and Africa. J. Med. Entomol. 15, 307–417 (1979). 113533 10.1093/jmedent/15.4.307

[b8] Gonzalez J. P., Cornet J. P., Wilson M. L. & Camicas J. L. Crimean-Congo haemorrhagic fever virus replication in adult Hyalomma truncatum and Amblyomma variegatum ticks. Res. Virol. 142, 483–488 (1991). 1803413 10.1016/0923-2516(91)90071-a

[b9] Logan T. M. *et al.* Replication of Crimean-Congo hemorrhagic fever virus in four species of ixodid ticks (Acari) infected experimentally. J. Med. Entomol. 27, 537–542 (1990). 2117664 10.1093/jmedent/27.4.537

[b10] Turell M. J. In Crimean-Congo Hemorrhagic Fever 143–154 (2007).

[b11] Nuttall P. A., Jones L. D. & Davies C. R. The role of arthropod vectors in arbovirus evolution. Adv. Dis. Vector Res. 8, 15–45 (1991).

[b12] Carroll S. A., Bird B. H., Rollin P. E. & Nichol S. T. Ancient common ancestry of Crimean-Congo hemorrhagic fever virus. Mol. Phylogenet. Evol. 55, 1103–1110 (2010). 20074652 10.1016/j.ympev.2010.01.006

[b13] Dohm D. J., Logan T. M., Linthicum K. J., Rossi C. a. & Turell M. J. Transmission of Crimean-Congo hemorrhagic fever virus by Hyalomma impeltatum (Acari:Ixodidae) after experimental infection. J. Med. Entomol. 33, 848–851 (1996). 8840695 10.1093/jmedent/33.5.848

[b14] Dickson D. L. & Turell M. J. Replication and tissue tropisms of Crimean-Congo hemorrhagic fever virus in experimentally infected adult Hyalomma truncatum (Acari: Ixodidae). J. Med. Entomol. 29, 767–773 (1992). 1404255 10.1093/jmedent/29.5.767

[b15] Lauring A. S. & Andino R. Quasispecies theory and the behavior of RNA viruses. PLoS Pathogens 6, 1–8 (2010). 10.1371/journal.ppat.1001005 PMC2908548 20661479

[b16] Ciota A. T. & Kramer L. D. Insights into arbovirus evolution and adaptation from experimental studies. Viruses 2, 2594–2617 (2010). 21994633 10.3390/v2122594 PMC3185588

[b17] Forrester N. L., Coffey L. L. & Weaver S. C. Arboviral bottlenecks and challenges to maintaining diversity and fitness during mosquito transmission. Viruses 6, 3991–4004 (2014). 25341663 10.3390/v6103991 PMC4213574

[b18] Deardorff E. R. *et al.* West nile virus experimental evolution *in vivo* and the trade-off hypothesis. PLoS Pathog. 7 (2011). 10.1371/journal.ppat.1002335 PMC3213084 22102808

[b19] Ciota A. T., Payne A. F., Ngo K. A. & Kramer L. D. Consequences of *in vitro* host shift for St. Louis encephalitis virus. J. Gen. Virol. 95, 1281–1288 (2014). 24643879 10.1099/vir.0.063545-0 PMC4027038

[b20] Bente D. a. *et al.* Pathogenesis and immune response of Crimean-Congo hemorrhagic fever virus in a STAT-1 knockout mouse model. J. Virol. 84, 11089–11100 (2010). 20739514 10.1128/JVI.01383-10 PMC2953203

[b21] Brackney D. E., Brown I. K., Nofchissey R. A., Fitzpatrick K. A. & Ebel G. D. Homogeneity of Powassan virus populations in naturally infected Ixodes scapularis. Virology 402, 366–371 (2010). 20434750 10.1016/j.virol.2010.03.035 PMC2875267

[b22] Weaver S. C. Evolutionary influences in arboviral disease. Current Topics in Microbiology and Immunology 299, 285–314 (2006). 16568903 10.1007/3-540-26397-7_10 PMC7120121

[b23] Ikegami T. Molecular biology and genetic diversity of Rift Valley fever virus. Antiviral Research 95, 293–310 (2012). 22710362 10.1016/j.antiviral.2012.06.001 PMC3586937

[b24] Duarte E., Clarke D., Moya A., Domingo E. & Holland J. Rapid fitness losses in mammalian RNA virus clones due to Muller’s ratchet. Proc. Natl. Acad. Sci. USA 89, 6015–6019 (1992). 1321432 10.1073/pnas.89.13.6015 PMC402129

[b25] Elena S. F. *et al.* Evolution of fitness in experimental populations of vesicular stomatitis virus. Genetics 142, 673–679 (1996). 8849878 10.1093/genetics/142.3.673 PMC1207009

[b26] Escarmis C. *et al.* Genetic lesions associated with Muller’s ratchet in an RNA virus. J. Mol. Biol. 264, 255–267 (1996). 8951375 10.1006/jmbi.1996.0639

[b27] Escarmís C., Dávila M. & Domingo E. Multiple molecular pathways for fitness recovery of an RNA virus debilitated by operation of Muller’s ratchet. J. Mol. Biol. 285, 495–505 (1999). 9878424 10.1006/jmbi.1998.2366

[b28] Yuste E., López-Galíndez C. & Domingo E. Unusual distribution of mutations associated with serial bottleneck passages of human immunodeficiency virus type 1. J. Virol. 74, 9546–9552 (2000). 11000225 10.1128/jvi.74.20.9546-9552.2000 PMC112385

[b29] Yuste E., Sánchez-Palomino S., Casado C., Domingo E. & López-Galíndez C. Drastic fitness loss in human immunodeficiency virus type 1 upon serial bottleneck events. J. Virol. 73, 2745–2751 (1999). 10074121 10.1128/jvi.73.4.2745-2751.1999 PMC104031

[b30] Escarmís C., Lázaro E., Arias A. & Domingo E. Repeated Bottleneck Transfers Can Lead to Non-cytocidal Forms of a Cytopathic Virus: Implications for Viral Extinction. J. Mol. Biol. 376, 367–379 (2008). 18158159 10.1016/j.jmb.2007.11.042

[b31] Weaver S. C., Brault a. C., Kang W. & Holland J. J. Genetic and fitness changes accompanying adaptation of an arbovirus to vertebrate and invertebrate cells. J. Virol. 73, 4316–4326 (1999). 10196330 10.1128/jvi.73.5.4316-4326.1999 PMC104213

[b32] Smith D. R., Adams A. P., Kenney J. L., Wang E. & Weaver S. C. Venezuelan equine encephalitis virus in the mosquito vector Aedes taeniorhynchus: Infection initiated by a small number of susceptible epithelial cells and a population bottleneck. Virology 372, 176–186 (2008). 18023837 10.1016/j.virol.2007.10.011 PMC2291444

[b33] Kenney J. L., Adams A. P., Gorchakov R., Leal G. & Weaver S. C. Genetic and anatomic determinants of enzootic Venezuelan equine encephalitis virus infection of Culex (Melanoconion) taeniopus. PLoS Negl. Trop. Dis. 6 (2012). 10.1371/journal.pntd.0001606 PMC3317907 22509419

[b34] Forrester N. L., Guerbois M., Seymour R. L., Spratt H. & Weaver S. C. Vector-Borne Transmission Imposes a Severe Bottleneck on an RNA Virus Population. PLoS Pathog. 8 (2012). 10.1371/journal.ppat.1002897 PMC3441635 23028310

[b35] Weidmann M. *et al.* Quantitative analysis of particles, genomes and infectious particles in supernatants of haemorrhagic fever virus cell cultures. Virol. J. 8, 81 (2011). 21349180 10.1186/1743-422X-8-81 PMC3056813

[b36] Stauffer Thompson K. A. & Yin J. Population dynamics of an RNA virus and its defective interfering particles in passage cultures. Virol. J. 7, 257 (2010). 20920247 10.1186/1743-422X-7-257 PMC2955718

[b37] Höper D., Hoffmann B. & Beer M. A comprehensive deep sequencing strategy for full-length genomes of influenza A. PLoS One 6 (2011). 10.1371/journal.pone.0019075 PMC3084732 21559493

[b38] Beck A. *et al.* Comparison of the live attenuated yellow fever vaccine 17D-204 strain to its virulent parental strain asibi by deep sequencing. J. Infect. Dis. 209, 334–344 (2014). 24141982 10.1093/infdis/jit546 PMC3883177

[b39] Parameswaran P. *et al.* Genome-Wide Patterns of Intrahuman Dengue Virus Diversity Reveal Associations with Viral Phylogenetic Clade and Interhost Diversity. J. Virol. 86, 8546–8558 (2012). 22647702 10.1128/JVI.00736-12 PMC3421746

[b40] Nuttall P. A., Jones L. D., Labuda M. & Kaufman W. R. Adaptations of arboviruses to ticks. J. Med. Entomol. 31, 1–9 (1994). 8158611 10.1093/jmedent/31.1.1

[b41] Booth T. F., Steele G. M., Marriott A. C. & Nuttall P. A. Dissemination, replication, and trans-stadial persistence of Dugbe virus (nairovirus, bunyaviridae) in the tick vector Amblyomma variegatum. Am. J. Trop. Med. Hyg. 45, 146–157 (1991). 1867347 10.4269/ajtmh.1991.45.146

[b42] Bergeron E. *et al.* Recovery of Recombinant Crimean Congo Hemorrhagic Fever Virus Reveals a Function for Non-structural Glycoproteins Cleavage by Furin. PLoS Pathog. 11, e1004879 (2015). 25933376 10.1371/journal.ppat.1004879 PMC4416775

[b43] Ozkaya E. *et al.* Molecular epidemiology of Crimean-Congo hemorrhagic fever virus in Turkey: Occurrence of local topotype. Virus Res. 149, 64–70 (2010). 20079776 10.1016/j.virusres.2009.12.014

[b44] Casati S., Gern L. & Piffaretti J.-C. Diversity of the population of Tick-borne encephalitis virus infecting Ixodes ricinus ticks in an endemic area of central Switzerland (Canton Bern). J. Gen. Virol. 87, 2235–2241 (2006). 16847119 10.1099/vir.0.81783-0

[b45] Goedhals D., Bester P. A., Paweska J. T., Swanepoel R. & Burt F. J. Next-generation sequencing of southern African Crimean-Congo haemorrhagic fever virus isolates reveals a high frequency of M segment reassortment. Epidemiol. Infect. 142, 1952–1962 (2014). 24786748 10.1017/S0950268814000818 PMC9151272

[b46] Honig J. E., Osborne J. C. & Nichol S. T. The high genetic variation of viruses of the genus Nairovirus reflects the diversity of their predominant tick hosts. Virology 318, 10–16 (2004). 14972529 10.1016/j.virol.2003.09.021

[b47] Lobo F. P. *et al.* Virus-host coevolution: Common patterns of nucleotide motif usage in Flaviviridae and their hosts. PLoS One 4 (2009). 10.1371/journal.pone.0006282 PMC2707012 19617912

[b48] Brackney D. E., Beane J. E. & Ebel G. D. RNAi targeting of West Nile virus in mosquito midguts promotes virus diversification. PLoS Pathog. 5 (2009). 10.1371/journal.ppat.1000502 PMC2698148 19578437

[b49] Aljamali M. N., Sauer J. R. & Essenberg R. C. RNA interference: Applicability in tick research. in Experimental and Applied Acarology 28, 89–96 (2002). 14570119 10.1023/a:1025346131903

[b50] Won S., Ikegami T., Peters C. J. & Makino S. NSm protein of Rift Valley fever virus suppresses virus-induced apoptosis. J. Virol. 81, 13335–13345 (2007). 17913816 10.1128/JVI.01238-07 PMC2168885

[b51] Crabtree M. B. *et al.* Infection and transmission of rift valley fever viruses lacking the NSs and/or NSm genes in mosquitoes: Potential role for NSm in mosquito infection. PLoS Negl. Trop. Dis. 6 (2012). 10.1371/journal.pntd.0001639 PMC3341344 22563517

[b52] Wölfel R. *et al.* Virus detection and monitoring of viral load in Crimean-Congo hemorrhagic fever virus patients. Emerg. Infect. Dis. 13, 1097–1100 (2007). 18214191 10.3201/eid1307.070068 PMC2878241

[b53] Langmead B. & Salzberg S. L. Fast gapped-read alignment with Bowtie 2. Nat Methods 9, 357–359 (2012). 22388286 10.1038/nmeth.1923 PMC3322381

[b54] Sharp P. M. & Li W. H. Codon usage in regulatory genes in Escherichia coli does not reflect selection for ‘rare’codons. Nucleic Acids Res. 14, 7737–7749 (1986). 3534792 10.1093/nar/14.19.7737 PMC311793

[b55] Tai V., Poon A. F. Y., Paulsen I. T. & Palenik B. Selection in coastal Synechococcus (cyanobacteria) populations evaluated from environmental metagenomes. PLoS One 6 (2011). 10.1371/journal.pone.0024249 PMC3170327 21931665

[b56] Morelli M. J. *et al.* Evolution of foot-and-mouth disease virus intra-sample sequence diversity during serial transmission in bovine hosts. Vet. Res. 44 (2013). 10.1186/1297-9716-44-12 PMC3630017 23452550

