# Supplementary material for: Transstadial Transmission and Long-term Association of Crimean-Congo Hemorrhagic Fever Virus in Ticks Shapes Genome Plasticity
Source: Sci Rep. 2016 Oct 24;6:35819. doi: 10.1038/srep35819 (PMC5075774; doi:10.1038/srep35819)
Supplement: Supplementary Information [file srep35819-s1.doc]

**Supplementary information**

**Title: Transstadial Transmission and Long-term Association of Crimean-Congo Hemorrhagic Fever Virus in Ticks Shapes Genome Plasticity**

Authors: Han Xia, Andrew S. Beck, Aysen Gargili, Naomi Forrester, Alan D.T. Barrett & Dennis A. Bente


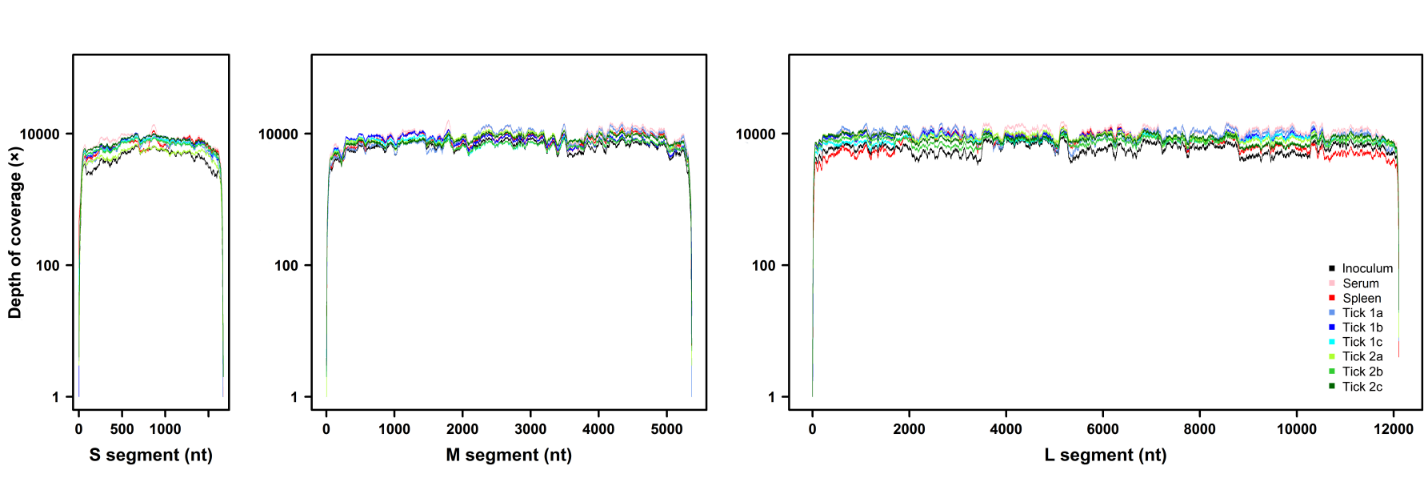


**Fig S1. Coverage of the genome obtained with filtered, removed duplicated reads.**

After removed duplicated reads, 2.76, 4.31, 3.64, 4.82, 4.70, 6.63, 5.73, 5.52, and 5.55 million high quality reads were produced for each sample (inoculum, serum, spleen, tick 1a, tick 1b, tick 1c, tick 2a, tick 2b, tick 2c). And all samples received generous coverage for each segment. 4300× for S, 6300× for M, and 5800× for L segment.

**Table S2. Verified and proposed functional domains used for calculating dN/dS**

| **Segment** | **Proposed functional domain** | **Amino acid region** |
| --- | --- | --- |
| **S** | Weaker RNA Binding Domain | 1-160 |
|  | Strong RNA Binding Domain | 240-482 |
| **M** | Mucin-Like domain | 23-243 |
|  | GP38 | 248-515 |
|  | Golgi targeting signal on Gn | 525-696 |
|  | Gn | 520-803 |
|  | NSm | 808-991 |
|  | Gc | 1040-1684 |
|  | Cystine Rich area | 1165-1208 |
|  | Fusion domain (Class II Fusion Protein) | 1191-1224 |
|  | Highly Conserved Epitope Binding Domain | 1214-1340 |
|  | Highly conserved neutralizing epitope | 1445-1566 |
| **L** | Ovarian Tumor-like Domain | 29-158 |
|  | Helicase/Gyrase | 570-670 |
|  | Polymerase Variable Region | 762-810 |
|  | Leucine Zipper | 1328-1407 |
|  | Potential endonuclease domain | 1867-2100 |
|  | RdRp Catalytic Site | 2269-2580 |
|  | Whole Polymerase Module | 2043-2710 |

**Table S3. Sample information**

| **Sample type** | **Group** | **Name** |
| --- | --- | --- |
| **Cell culture** | CCHFV IbAr 10200 stock | Inoculum |
| **Mammalian Host** | Mouse tissue | Serum |
|  |  | Spleen |
| **Tick vector** | Group 1, newly molted | Tick 1a |
|  |  | Tick 1b |
|  |  | Tick 1c |
|  | Group 2, 1 year old | Tick 2a |
|  |  | Tick 2b |
|  |  | Tick 2c |

**Table S4. Primer pairs used for CCHFV amplicons**

| **Segment** | **Forward (5’-3’)** | **Reverse(5’-3’)** | **Position** |
| --- | --- | --- | --- |
| **S** | SF: TCTCAAAGAAACACGTGCCGC | SR: TCTCAAAGATATCGTTGCCGC | 1-1,672 nt |
| **M** | M1F: TCTCAAAGAAATACTTGCGGCACGT | M1R: GTTTTCGCATAGCTTGAATGACCTC | 1-1,850 nt |
|  | M2F: AAGGACAGAGACTGCAGAGATCCAC | M2R: CCCTGATAAGTACTCGAAGACAGGAGA | 1,694-3,551 nt |
|  | M3F: CAGGCAGATCAGAATCAATTATGAAGC | M3R: TCTCAAAGATATAGTGGCGGCACGC | 3,400-5,366 nt |
| **L** | L1F: TCTCAAAGATATCAATCCCCCC | L1R: GTCTGTATCAACATGGTCAG | 1-2,079 nt |
|  | L2F: CAATGAGTGCAGGCAAGATTG | L2R: CTTGGCACAGAACACTTTGA | 1,680-3,741 nt |
|  | L3F: GATGATGAGCATGTCAGGCAT | L3R: GATGATGAGCATGTCAGGCAT | 3,478-5,307 nt |
|  | L4F: TGCCCAAATGTGAGGAAAGC | L4R: GAACAGCAGTGTATCGGGCC | 5,075-7,182 nt |
|  | L5F: AGCAAGGTCATATATGAGATG | L5R: TATTCTTCTGTCGAAACATTGA | 6,772-8,862 nt |
|  | L6F: TCATTTACAGCAGCAGAATG | L6R: TGGTATGTGGTCTGAGTATC | 8,462-10,570 nt |
|  | L7F: TCTTGGATGAGTAAGAGGAGGACT | L7R: TCTCAAAGAAATCGTTCCCCCCAC | 1,0200-12,160 nt |
